# Straight Channel Microfluidic Chips for the Study of Platelet Adhesion under Flow

**DOI:** 10.21769/BioProtoc.3195

**Published:** 2019-03-20

**Authors:** Alexander Dupuy, Lining Arnold Ju, Freda H Passam

**Affiliations:** 1 Heart Research Institute, Newtown, NSW 2042, Australia; 2 University of Sydney, Camperdown, NSW 2006, Australia

**Keywords:** Microfluidics, Biochip, Platelet, Thrombosis, Biorheology

## Abstract

Microfluidic devices have become an integral method of cardiovascular research as they enable the study of shear force in biological processes, such as platelet function and thrombus formation. Furthermore, microfluidic chips offer the benefits of *ex vivo* testing of platelet adhesion using small amounts of blood or purified platelets. Microfluidic chips comprise flow channels of varying dimensions and geometries which are connected to a syringe pump. The pump draws blood or platelet suspensions through the channel(s) allowing for imaging of platelet adhesion and thrombus formation by fluorescence microscopy. The chips can be fabricated from various blood-compatible materials. The current protocol uses commercial plastic or in-house polydimethylsiloxane (PDMS) chips. Commercial biochips offer the advantage of standardization whereas in-house chips offer the advantage of decreased cost and flexibility in design. Microfluidic devices are a powerful tool to study the biorheology of platelets and other cell types with the potential of a diagnostic and monitoring tool for cardiovascular diseases.

## Background

Platelets primary function is to maintain blood in circulation by sealing off any breach of vessel integrity which would otherwise lead to exsanguination. For this function, platelets have evolved specialized cell surface receptors to allow adhesion to the endothelium and subendothelium at varying shear rates, ranging from 200 s^-1^ in venous circulation to > 1 x 10^4^ s^-1^ in stenotic arteries (Chatzizisis *et al.*, 2008). Shear stress is implicated in the development of atherosclerotic lesions (Chatzizisis *et al.*, 2008). Shear rate and stress impact protein expression (Morigi *et al.*, 1995) and function (Yago *et al.*, 2004; Ju *et al.*, 2013 and 2015). Hence, the effect of flow on cell function must be included in vascular research.

Flow chambers, introduced in 1973 by Baumgartner (Baumgartner, 1973), incorporate the parameters of shear rate, which is the velocity gradient throughout a moving fluid, and shear stress, which is the force experienced by the wall of a conductance vessel due to the friction force of a moving fluid. The application of microfluidics has enabled significant discoveries in platelet biology. For example, by perfusing blood under controlled shear rates over adhesive proteins, it was found that platelets utilize their receptor glycoprotein Ib alpha (GPIbα) to adhere to von Willebrand factor (vWF), whereas, they use their receptor integrin alpha IIb beta 3 (α_IIb_β_3_) to adhere to fibrinogen (Ruggeri, 2009; Ju *et al.*, 2018; Passam *et al.*, 2018). Under low-intermediate shear rates (< 1 x 10^3^ s^-1^), typically found in veins and large arteries, platelet adhesion is predominately mediated by integrin α_IIb_β_3_. Under high shear rates (1 x 10^3^-1 x 10^4^ s^-1^), typically found in arterial microcirculation and in arterial stenosis, platelet adhesion is predominantly vWF-dependent (Reininger *et al.*, 2006; Ruggeri *et al.*, 2006; Jackson, 2007). Because of the effect of fluid dynamics on cell function and protein expression, microfluidics is a powerful tool to study cells in circulation.

Commercial microfluidic devices have been developed. The rheology of biological fluids passing through these devices has been well characterized (Lane *et al.*, 2012). However, these devices are limited by high cost and inflexible geometry designs. Recent studies in the field have utilized polydimethylsiloxane (PDMS) elastomers to fabricate microfluidic flow chambers, or ‘biochips’, with typical channel geometries of 10-1,000 μm in x and y-axes and 10-200 μm in the z-axis. PDMS is a transparent and elastic material which has been widely used to create microfluidic channels with various geometries such as stenosis (Nesbitt *et al.*, 2009; Tovar-Lopez *et al.*, 2013), bifurcation (Tsai *et al.*, 2012), aneurysm (Mannino *et al.*, 2015), and spiral channels for two-dimensional cell sorting (Hou *et al.*, 2016). In contrast to the conventional flow chamber that usually requires milli-liter sample volumes, the microfluidic biochip only requires micro-liter samples, making it a perfect analytical tool for small volumes (*e.g.*, pediatric or rare samples and mouse studies). In addition, the biochips described in this protocol are reusable, making them an economic and versatile choice for microfluidic studies.

Using microfluidics, we have recently shown that a member of the thiol isomerase family, endoplasmic reticulum protein 5 (ERp5) regulates platelet adhesion to fibrinogen in a shear-dependent manner (Passam *et al.*, 2018). The current protocol describes the fabrication and assembly of an in-house straight-channel biochip. The protocol also describes the application of straight-channel commercial and in-house chips for the study of platelet adhesion under controlled shear conditions.

## Materials and Reagents

1. 2 ml glass Luer lock syringe (Tömopal, catalog number: 140-1502)
2. 24 mm x 50 mm x 0.17 mm #1 borosilicate rectangular coverslips (Thermo Fisher, catalog number: MENCS24501GP)
3. 150 mm x 20 mm Petri dish (SARSTEDT, catalog number: 82.1184.500)
4. Medical grade Tygon tubing, 0.8 mm ID and 1.6 mm OD (Watson-Marlow)
5. 20 ml Syringe Luer Lok Tip (BD, catalog number: 302830)
6. Silicon wafer, P-type boron dope, 4 inches (Research and Prototype Foundry, University of Sydney Nano Institute)
7. 365 nm UV lamp (Research and Prototype Foundry, University of Sydney Nano Institute)
8. 1.5 ml transfer pipettes (Thermo Fisher, catalog number: 282TS)
9. Connecta^TM^ 3-way stopcock, 2 female 1 rotating male Luer lock connector (BD, catalog number: 394995)
10. Venous blood collection set (BD, Vacutainer Safety-Lok Blood Collection Set with Pre-Attached Holder, 21 G, catalog number: 368654)
11. Scotch tape
12. Whole Blood Tube w/ Acid Citrate Dextrose (ACD) Sol A (BD, catalog number: 364606)
13. Acid Citrate Dextrose (store at 4 °C, shelf-life: 6 months) (Sigma-Aldrich, catalog number: C3821-50ML)
14. Bovine serum albumin (store dessicated albumin at 4 °C, shelf-life: 6 months) (Sigma-Aldrich, catalog number: A8531-1VL)
15. Butan-1-ol (Ajax Finechem, catalog number: AJA107-2.5LGL)
16. Calcein AM (store at -20 °C, shelf-life: 6 months) (Thermo Fisher, catalog number: C1430)
17. Rat anti-mouse glycoprotein Ib antibody conjugated to Dylight fluor 488 (Emfret Analytics, catalog number: X488)
18. DMSO, store in the dark at room temperature (Life Technologies, catalog number: D12345)
19. Extran^®^ MA 02, store at room temperature (Merck, catalog number: 1075532500)
20. Fibrinogen, 4 mg/ml (store at -80 °C, shelf-life: 12 months) (Haematologic Technologies, catalog number: HCI-0150R)
21. Von Willebrand Factor (vWF), 0.3 mg/ml (store at -80 °C, shelf-life: 12 months) (Haematologic Technologies, catalog number: HCVWF-0190)
22. Photoresist SU-8 2000 (MicroChem, catalog number: SU-8 2050)
23. Photoresist Developer Microposit Thinner Type P (Rohm and Haas)
24. Isopropanol (IPA) (Sigma-Aldrich, catalog number: W292907)
25. Edge bead remover (MicroChem, EBR PG)
26. ddH_2_O
27. Sylgard(R) 184 Silicone Elastomer, store at room temperature (polydimethyl siloxane; PDMS) (Dow Corning, catalog number: 1317318)
28. Prostaglandin E1 (PGE1), 5 mg/ml (14 mM) in 100% ethanol (store at -80 °C, shelf-life: 6 months) (Sigma-Aldrich, catalog number: P5515-1MG)
29. HEPES (Sigma-Aldrich, catalog number: H3375-100G)
30. NaCl (Sigma-Aldrich, catalog number: S7653-250G)
31. KCl (Sigma-Aldrich, catalog number: P933-500G)
32. Na_2_HPO_4_ (Sigma-Aldrich, catalog number: RES20908-A702X)
33. KH_2_PO4 (Sigma-Aldrich, catalog number: P0662-500G)
34. NaHCO_3_ (Sigma-Aldrich, catalog number: S5761-500G)
35. MgCl_2 _(Sigma-Aldrich, catalog number: M8266-100G)
36. CaCl_2 _(Sigma-Aldrich, catalog number: C5670-100G)
37. D-glucose (Sigma-Aldrich, catalog number: G8270-100G)
38. 1x PBS (Phosphate Buffered Saline) (store at room temperature, shelf-life: 6 months) (see Recipes)
39. 10% Extran^®^ (store at room temperature, shelf-life: 1 year) (see Recipes)
40. Blocking buffer (store at 4 °C, shelf-life: 2 weeks) (see Recipes)
41. HEPES-Tyrode’s buffer with glucose, store HEPES-Tyrode’s without glucose at room temperature for 6 months, add glucose fresh each time (see Recipes)

## Equipment

- For in-house chips
  1. Centrifuge (Eppendorf, catalog number: 5810000017)
  2. Heidelberg tabletop maskless aligner (Heidelberg Instruments, catalog number: MLA100)
  3. Pipettes (Eppendorf, catalog number: 3120000917)
  4. 1 and 6 mm Harris Uni-Core Biopsy punch (World Precision Instruments, catalog numbers: 501907, 501910)
  5. Water bath (Ratek instruments, catalog number: WB20)
  6. Nalgene^®^ vacuum desiccator (Sigma-Aldrich, catalog number: D2797-1EA)
  7. Heratherm Gravity convection oven (Thermo Fisher, catalog number: 51028112)
  8. Hotplate (SAWATEC, catalog number: HP-150)
*Note: All hot plates used for this process are an integral part of our Ritetrack Equipment, which also contains the coater/developer modules used for this project.*
  9. PHD ULTRA^TM^ Programmable Infuse/Withdraw syringe pump (Harvard Apparatus, catalog number: 70-3007)
  10. Sonicator (Thermoline, catalog number: UB-405)
  11. Tabletop centrifuge (Thermo Fisher, catalog number: 75002415)
  12. Olympus fluorescent microscope IX81 60x oil immersion objective NA 1.35
- For commercial chips
  1. Inlet and Outlet pins (Cellix, catalog number: SS-P-B1IC-B1OC-PACK200)
  2. Tygon tubing for BiochipConnect (Cellix, catalog number: TUBING-TYGON-BIC-B1OCROLL100FT)
  3. Vena8 Fluoro+^TM^ biochips (Cellix, catalog number: V8CF-400-100-02P10)
  4. Mirus Evo Nanopump (Cellix)
  5. AxioObserver A1 Inverted Epi-Fluorescence microscope (Zeiss, Germany)
  6. ExiBlu CCD camera (Q imaging, Canada)
- For platelet separation
  1. Sysmex KX21 Hematology Analyzer (Sysmex America, Inc. Lincolnshire, Illinois, USA)

## Software

- For in-house chips
  1. Fiji/ImageJ 1.52h (https://fiji.sc/)
  2. GraphPad Prism (GraphPad software, https://www.graphpad.com/scientific-software/prism/)
- For commercial chips
  1. VenaFlux 2.3 imaging software
  2. ImagePro Premier 64-bit software image analysis
  3. GraphPad Prism (GraphPad software, https://www.graphpad.com/scientific-software/prism/)
*
Note: The VenaFlux 2.3 imaging software and ImagePro Premier 64-bit image analysis software are pre-installed in the PC of the Venaflux platform (Cellix Ltd., Unit 1, Longmile Business Park, Longmile Road, Dublin 12, Ireland, info@wearecellix.com).
*

## Procedure

- Fabrication of PDMS biochips
The microfluidic biochips can be fabricated with PDMS (Sylgard 184 kit) casted from a master mould on a silicon wafer by photolithography (Qin *et al.*, 2010). Most universities and institutes have photolithography and clean room facilities for the fabrication of the master mould. In this protocol, the photolithography is conducted at the Research and Prototype Foundry at University of Sydney Nano Institute. It is possible to perform photolithography without a foundry. The equipment required would include: 1. Air plasma system, 2. Oven for PDMS curing, 3. Desiccator for PDMS degassing, 4. Programmable spin coater, 5. UV lamp–LED exposure, 6. Programmable hot plate. We refer interested readers to: Microfluidic device design, fabrication, and testing protocols or Elveflow.
  1. Dehydrate a 4 in. silicon wafer at 200 °C for 20 min, then apply an adhesion promoter for 30 s at 120 °C.
  2. Spin coat the wafer with SU-8 2050 (high contrast, epoxy-based) photoresist using a spread cycle of 70 and 22 *× g* for 30 s and a development cycle of 1,000 and 0.5 *× g* for 30 s in order to achieve a desired film thickness in the z-axis (*e.g.*, 50 μm in this protocol).
  3. Conduct a cycle of edge bead removal for 30 s using edge bead removal solvent.
  4. Directly write the pattern to the SU-8 film using a dose of 365 nm UV light at 100 mJ/cm^2^.
  5. Crosslink the film pattern by baking on a hotplate and ramping the temperature at 5 °C/min, starting at 23 °C and holding at 90 °C to dry out the solvents. The ramping profile is achieved by proximity, plus vacuum contact bake steps, for a total duration of 805 s.
  6. Allow the film to cool on the hotplate to room temperature.
*Note: Keep the film on the hotplate to avoid thermal stress.*
  7. Develop unexposed SU-8 photoresist using fresh developer solution for 3.5 min in a rocker.
  8. Rinse the wafer with IPA.
  9. Dry the wafer using pressurized nitrogen.
  10. Hard-bake the wafer at 150 °C for 30 min.
*Note: Alternatively, follow* *the Permanent Epoxy Negative Photoresist Processing Guidline* *from MicroChem.*
*
Note: The following Steps (A11-A17) can be performed in the research lab (these steps do not require a clean room). (Figure 1)
*
  11. Transfer the silicon wafer into a 150 mm Petri dish, SU-8 side facing upwards, and secure with scotch tape.
  12. Mix the Sylgard 184 kit PDMS base with the kit curing agent at a ratio of 10:1 by weight. For example, to prepare 198 g of PDMS, mix 180 g of the PDMS base with 18 g of the curing agent.
*Notes:*
    - *Due to the viscosity of the base and curing agent, it is easier to prepare the kit by weight. It is important that the base and curing agent are mixed completely to prevent inconsistencies in the curing of the final product. Mix thoroughly until no visible streaks can be seen. This process will create a large amount of small bubbles that need to be removed by degassing.*
    - *Once the curing agent is added to the PDMS base, the PDMS will slowly begin to cure and harden at room temperature. This will be noticeable after 4 h. After 16 h, the PDMS will become too viscous to practically work with.*
  13. Pour the PDMS mixture over the pre-made mold, creating a 4-5 mm thick film.
  14. Place the PDMS and mold in a Nalgene^®^ vacuum desiccator and degas for 30 min.
*Note: The amount of time required to degas the mixture is dependent on the volume and surface area of PDMS. The mixture must be degassed completely, otherwise bubbles will form during the curing process which will change the morphology of the device. For 200 g of PDMS placed in a 100 mm diameter Petri dish, 2 h is sufficient to completely degas the mixture.*
  15. Bake (cure) the PDMS mixture and mold in the oven at 80 °C for 4 h.
  16. Cut out the cured PDMS chips from the mold gently and carefully.
*Notes:*
    - *For the design used in this protocol, the biochip can be cut from the mold and the remaining PDMS can be left on the mold to reduce the amount of PDMS required for fabrication in the future.*
    - *The typical PDMS cut-out block has a length of 4 cm, width of 2 cm and height of 5 mm. Avoid larger PDMS cut-outs as these can bend the coverslip and obscure the plane of focus during image acquisition.*
    - *Take care not to damage or crack the mold whilst cutting out the biochip. Gradually cut out the PDMS by making consecutive, linear cuts into the PDMS and gently lift the PDMS off from the silicon wafer.*
  17. With the channel side facing upward, use a 6 mm diameter biopsy punch to cut out a hole at one end of the channel. Punch another hole at the opposite end of the channel using a 1 mm diameter biopsy punch.
*Note: The 6 mm hole is used as a well to hold the sample for the microfluidics experiment once the biochip is placed on a coverslip. The 1 mm hole is used to connect the channel to the syringe pump.*
    **Figure 1.**
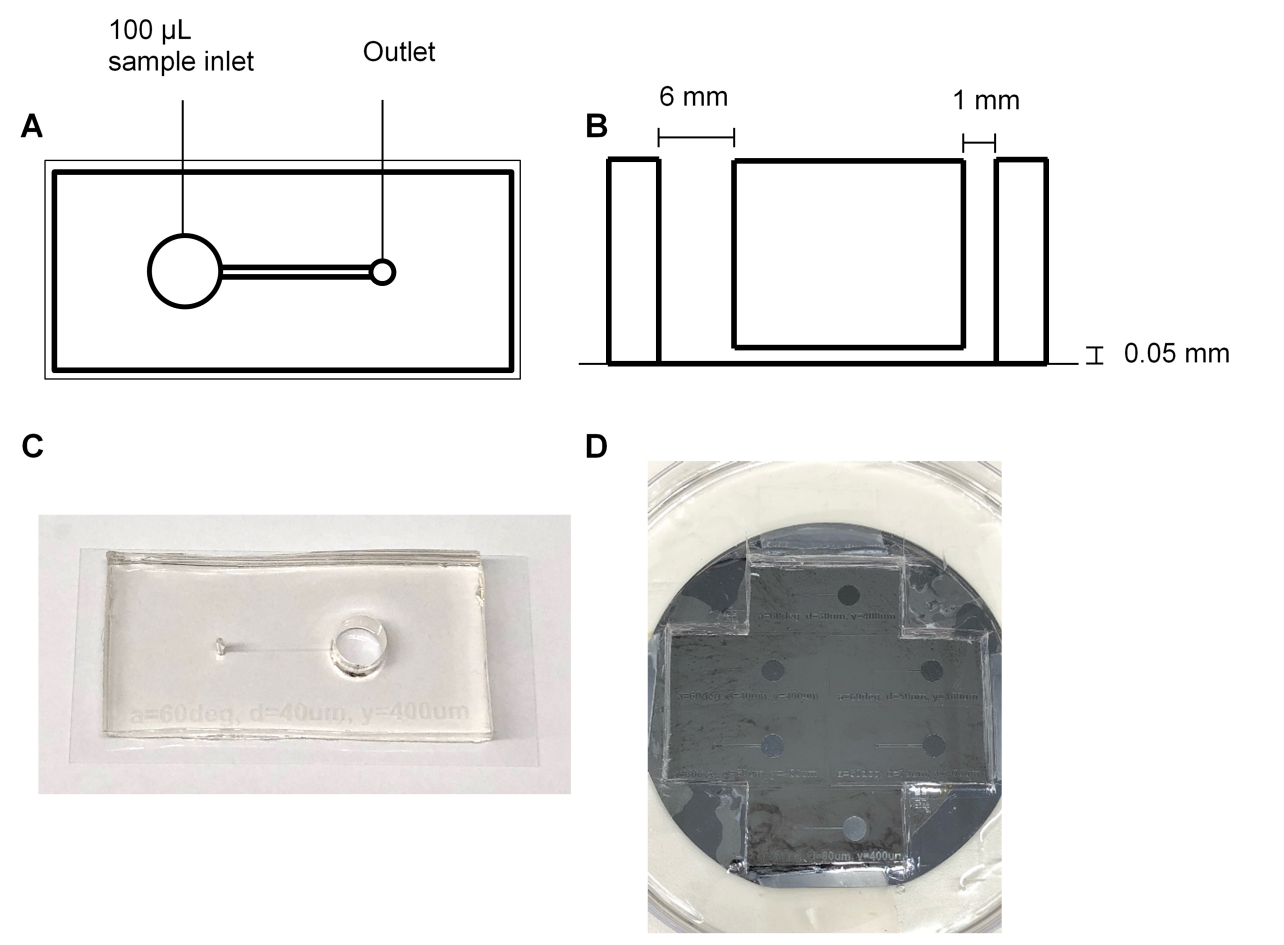
Fabrication of PDMS biochips. The PDMS biochip produced in this protocol features a 6 mm diameter inlet (well) which can hold up to 100 μl of sample, and a 1 mm diameter outlet which connects the channel to the pump. The channel has a length of 12 mm, width of 0.4 mm and a height of 0.05 mm (A, B, and C). The manufactured silicon wafer mask contains indentations which form the shape of the channel (D).
- Cleaning the biochip
  Dust and other impurities can affect the coating of coverslips with fibrinogen and obstruct the field of view under the microscope. Dust may also interfere with the adhesion of PDMS to the coverslip and the adhesion of platelets to the coated coverslip. The following is a gentle cleaning procedure to remove dust and impurities from the surface of PDMS and coverslips.
  1. Place the biochip in 400 ml of 10% extran and sonicate for 15 min.
  2. Blow dry the biochip completely using compressed air.
*Note: Make sure the biochip is completely dry before proceeding. Remnants from small droplets can compromise the contact between the biochip and the coverslip leading to leakage during a microfluidics experiment.*
  3. Place the biochip in 100% Butan-1-ol and sonicate for 30 min.
*
Note: The PDMS will swell slightly and show indentations after the Butan-1-ol treatment. This is normal and the PDMS will return to its original shape and size once sonicated in dH_2_O.
*
  4. Blow dry the biochip completely using compressed air. Place the biochip in 10% extran and sonicate for 10 min.
  5. Blow dry the device using compressed air. Place the biochip in dH_2_O and sonicate for 10 min.
  6. Blow dry the device using compressed air.
  7. With the channel side facing down, place the biochip on a #1 coverslip. Press the PDMS down on the coverslip with the palm of your hand and smooth out any bubbles with your finger.
*Notes:*
    - *A good seal needs to be made between the PDMS and the coverslip to prevent leakage of cells from the sides of the channel.*
    - *The coverslip should be cleaned to remove any dust and smudges that may interfere with contact between the PDMS and the coverslip. The coverslip can be cleaned using Steps B1 and B5.*
    - *The PDMS can be reused after the microfluidics experiment. Clean the PDMS again by following Procedure B. Store the PDMS in a sealed container away from light. The PDMS can be reused until it is permanently bent and does not stick properly to a coverslip. In our experience, the PDMS can be reused at least 10 times.*
    - *The size of the coverslip used depends on the size of the biochip. Choose a coverslip size that is closest to the area of the biochip.*
- Coating the channel of the biochip
The steps involved in the coating of the biochip (Section C) can be found in Video 1.
  1. Place 100 μl of fibrinogen (40 μg/ml final fibrinogen concentration in PBS, pH 7.4) in the 6 mm inlet of the biochip.
  2. Using a 1.5 ml transfer pipette, draw the fibrinogen through the channel and into the pipette.
*Notes:*
    - *A good seal between the transfer pipette and the 1 mm outlet must be made, otherwise the fibrinogen will not transverse the channel properly. Bubbles in the pipette indicate that good suction was not achieved.*
    - *Do not drain the inlet or the channel completely. Draw up enough of the fibrinogen solution until you are certain that the channel has been filled with the fibrinogen solution. To remove the pipette from the outlet, apply positive pressure on the transfer pipette until the fluid stops drawing into the pipette, then remove the pipette.*
    - *To check that the channel has been filled with the coating substrate (fibrinogen), rotate the biochip back and forth under light. Due to the refractive index mismatch between air and PDMS, an empty channel will be slightly less transparent than a channel filled with fluid, leading to a shimmering of the channel under light.*
  3. Incubate the biochip for 2 h at 25 °C.
*Note: The incubation period and temperature will depend on the coating substrate used to coat the channel. However, the longer the channel is left incubating, the greater the risk of the channel leaking.*
  4. Remove the fibrinogen in the inlet well, replace with 100 μl of Tyrode’s buffer or PBS and repeat Step D2 to wash the channel.
  5. Remove the Tyrode’s buffer or PBS from the inlet well and replace with 100 μl of blocking buffer. Repeat Step D2 to block the channel with blocking buffer. Incubate for 30 min at 25 °C.
  6. Remove the blocking buffer from the inlet well, replace with 100 μl of Tyrode’s buffer or PBS and repeat Step D2 to wash the channel.
*Note: Once washed, keep the Tyrode’s buffer or PBS in the channel until image acquisition. This will prevent the formation of air bubbles in the channel while flowing cells through the biochip.*
    **Video 1.**
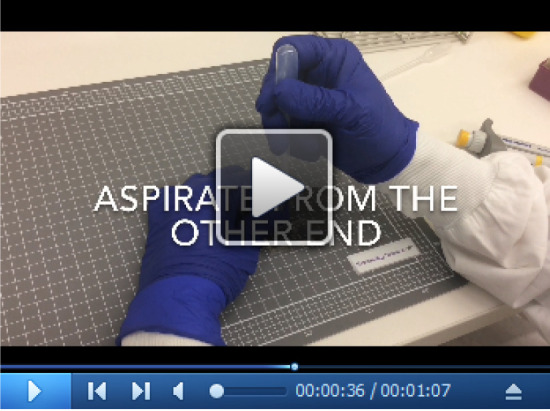
Coating of the in-house PDMS biochip
- Isolation of platelets from whole blood
***Human platelets***
  The procedure of drawing human blood by venipuncture is determined by the Institution’s Ethics and Protocols for human blood sampling.
  1. Draw 8 ml venous blood by venipuncture into an ACD tube.
  2. Centrifuge at 200 *× g* for 20 min, no brake, and separate the platelet-rich plasma (supernatant).
  3. Allow platelet-rich plasma to rest in the water bath at 37 °C for 30 min.
  4. Add PGE1 to the platelet rich plasma (1 µM final concentration) immediately prior to centrifugation.
  5. Centrifuge at 800 *× g* for 20 min, no brake, and discard the platelet-poor plasma (supernatant).
  6. Resuspend the platelet pellet in HEPES-Tyrodes’ buffer with glucose. Perform a platelet count on the hematology analyzer and adjust the platelet concentration to 3 x 10^5^/µl (Nesbitt *et al.*, 2009). Five minutes before perfusing through the microfluidic channel, add calcein to a final concentration of 1 µg/ml and keep the platelets in the dark until use. Use the labeled platelets within an hour as the calcein is effluxed out of the cell and cell fluorescence will be lost.
***Mouse platelets***
The procedure of drawing mouse blood by venipuncture is determined by the Institution’s Animal Ethics and local protocols.
  1. Draw 1 ml of blood by cardiac puncture after CO_2_ euthanization or by venipuncture of the inferior vena cava under anesthesia.
  2. Centrifuge the blood on a tabletop centrifuge at 400 *× g* for 5 min.
  3. Collect the platelet-rich plasma (supernatant) and 1/8 of the upper red blood cell layer.
  4. Add 200 µl of HEPES-Tyrodes’ buffer with glucose to the red blood cell layer and gently mix. Centrifuge the red blood cell layer at 400 *× g* for 5 min.
  5. Collect the supernatant and 1/8 of the upper red blood cell layer and pool with the platelet-rich plasma from Step 3.
  6. Centrifuge the pooled platelet-rich plasma at 200 *× g* for 6 min.
  7. Collect the platelet-rich plasma (supernatant) without disturbing the red blood cell layer.
  8. Add 200 µl of HEPES-Tyrodes’ buffer with glucose to the red blood cell layer and mix. Centrifuge the red blood cell layer at 200 *× g* for 6 min.
  9. Collect the supernatant without disturbing the red blood cell layer and pool with the platelet-rich plasma from Step 7.
  10. Allow the platelet-rich plasma to rest for 10 min at room temperature. At the end of the incubation, add PGE1 (0.5 µM final concentration) and centrifuge the plasma at 500 *× g* for 10 min.
  11. Discard the platelet-poor plasma (supernatant) and resuspend the pellet in HEPES-Tyrode’s buffer with glucose. Perform a platelet count on the hematology analyzer and adjust the platelet concentration to 300 x 10^3^/µl.
  12. Prior to performing a microfluidics assay, incubate the platelets with anti-GPIb 488 antibody (3 µg/ml final concentration) for 10 min at room temperature, in the dark.
*Notes: For both human and mouse platelets:*
  - *All centrifugation steps are at room temperature.*
  - *Platelet suspensions cannot be stored at 4 °C at any step.*
  - *Platelets should be used within 4 h of collection by venipuncture.*
  - *Perfusion assays are performed in the dark.*
- Image acquisition
  1. Assemble the syringe pump as shown below (Figure 2). Connect the 2 ml Luer lock glass syringe to the horizontal facing female Luer lock on the 3-way connector. Slip the silicone tubing onto the male Luer lock and screw the rotating lock over the tubing. Connect a water-filled 20 ml plastic Luer lock syringe to the final female Luer lock and place the glass syringe on the PHD syringe pump. Secure the glass syringe in place.
    **Figure 2.**
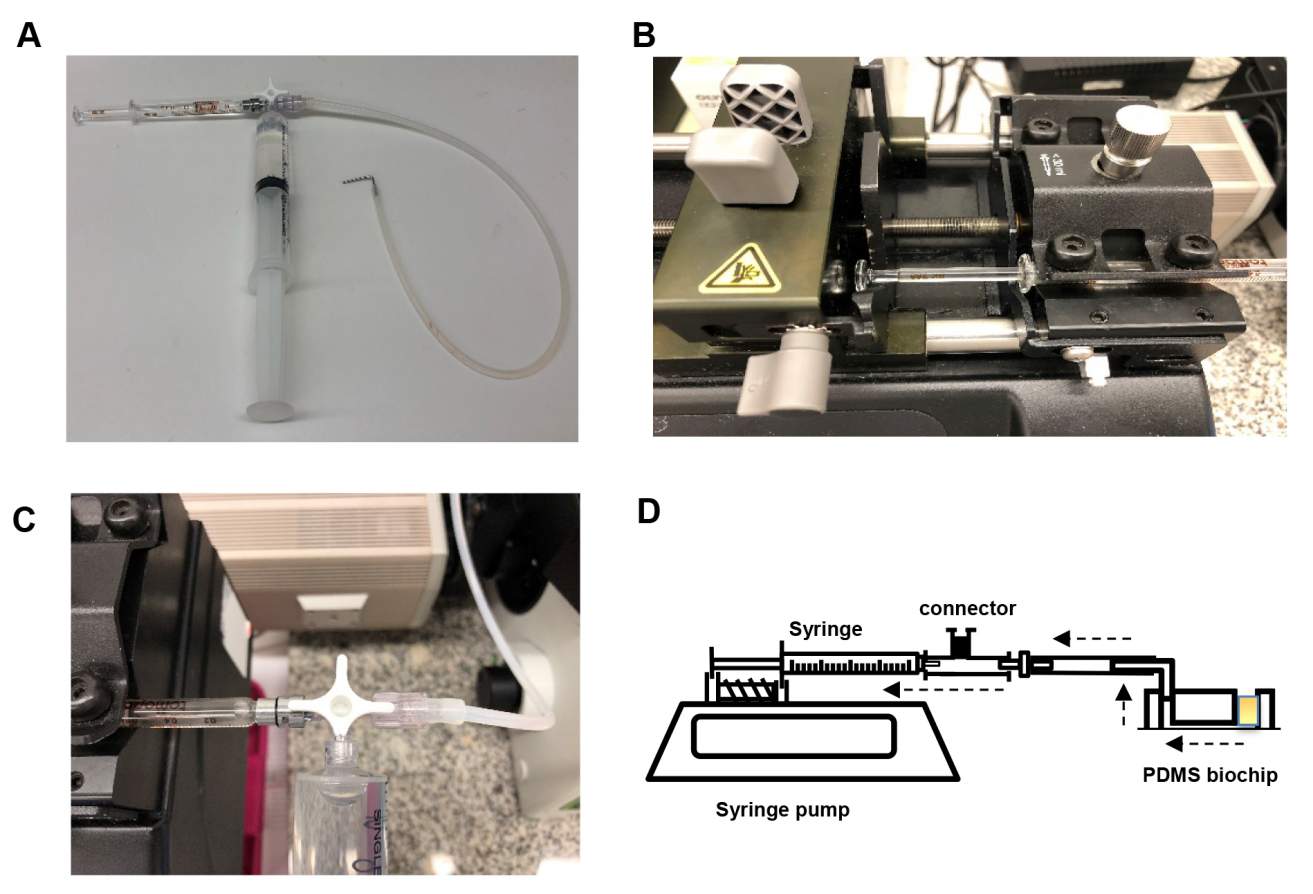
Setup of the syringe pump. A. Assembly of the 2 ml glass syringe, plastic 20 ml syringe filled with water (to flush the tubing after each experiment) and tubing (with a 1 mm diameter metal adapter) on a 3-way stopcock connector. B. Assembly of glass syringe on the pump. C. Rotation of the stopcock to enable flow from tubing to glass syringe. D. Schematic of the microfluidic chip set-up. Dashed arrows indicate the direction of flow. The yellow square in the chip represents the platelet suspension.
  2. Bend a 30 mm length piece of 1 mm diameter steel tubing in half at a 90° angle and insert the other end into medical grade Tygon tubing. Insert the metal tubing into the 1 mm hole outlet of the biochip (Figure 3). Secure the biochip onto the stage of the microscope and bring the plane of the coverslip into focus (Figure 4).
*
Note: The steps involved in the assembly of the biochip to the microscope (Figures 2-3) can be found in Video 2.
*
    **Video 2.**
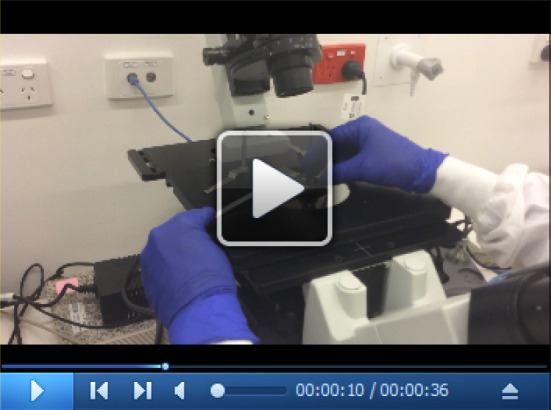
Assembly of the in-house PDMS biochip onto a microscope stage
    **Figure 3.**
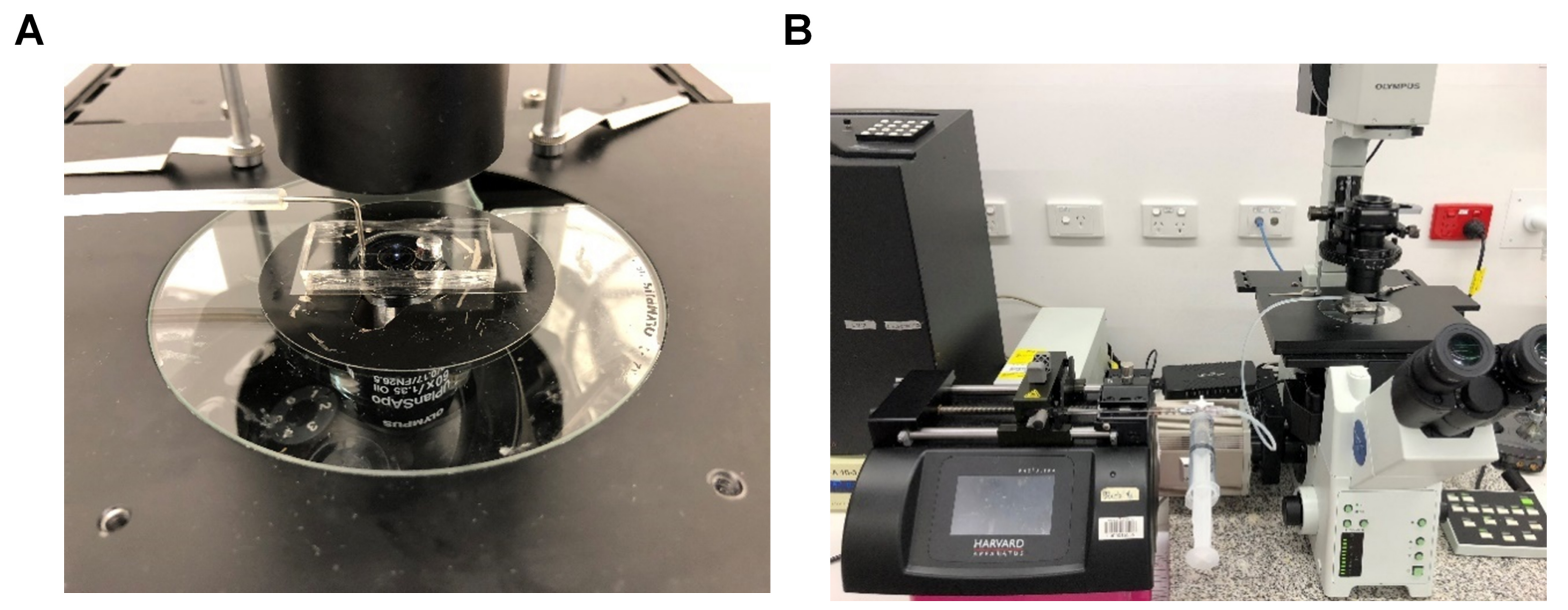
Final assembly of the microfluidics system. A. The 1 mm diameter metal tubing is connected to the 1 mm hole of the PDMS biochip. B. The system is then assembled to an inverted microscope.
    **Figure 4.**
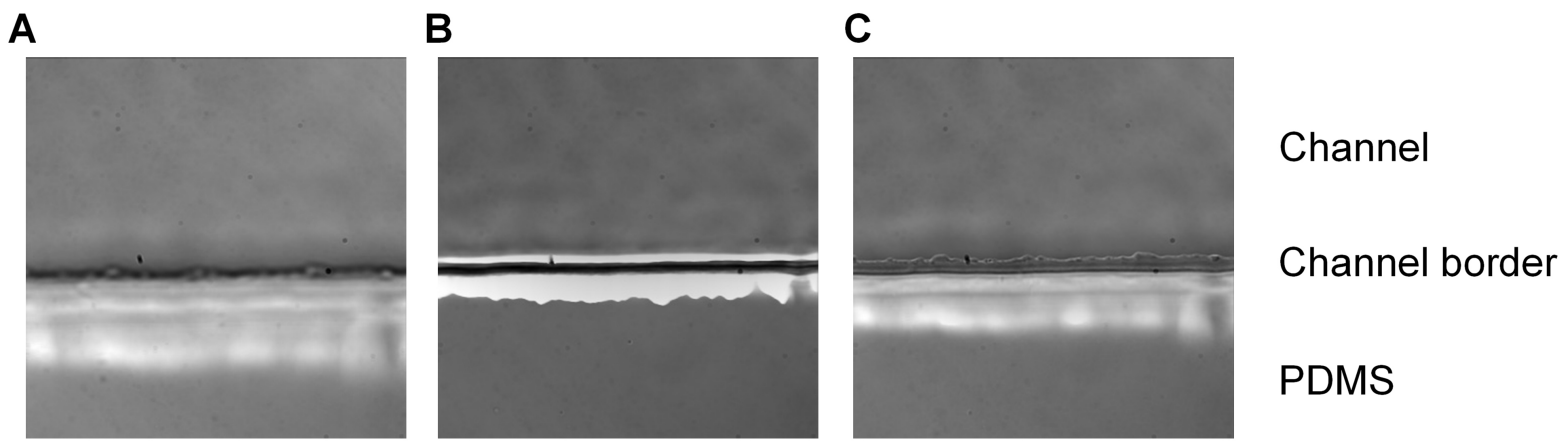
Planes of focus. Imaging of the biochip placed under an Olympus fluorescent microscope using a 60x objective. The plane of focus is displayed below the coverslip (A), above the coverslip (B) and on the coverslip (C). When the coverslip is in focus, the jagged edge of the PDMS (an artifact of the fabrication process) can be seen within the channel.
  3. Empty the inlet well and start the acquisition. Acquire a time-stack for both the DIC channel and fluorescent channel (excitation: 488 nm, emission: 520 nm) for ve+ stained platelets (Figure 5), setting the acquisition parameters to take images at regular time intervals.
  4. Place 100 μl of 3 x 10^5^μl washed platelets in the inlet well of the biochip.
  5. Start the syringe pump and draw fluid through the channel at a rate of 5 μl/min (500 s^-1) ^for 5 min. Alternatively, to expose the platelets to other shear rate and stress levels, use the simplified formulas (1, 2) below to calculate the flow rate needed. A table has been provided as a quick reference to calculate shear rates from flow rates for the chip used in this protocol (Table 1).
$Q=Wh^{2}\dot{\gamma }\;\times 10000$                     (1)
    where,
Q = flow rate (μl/min)
W = width of the channel (cm)
h = height of the channel (cm)
$\dot{\gamma }$ = desired shear rate (s^-1^)
$Wall\;shear\;stress\;\tau =\frac{6\mu Q}{Wh^{2}}$       (2)
    where,
W = width of the channel (cm)
h = height of the channel (cm)
μ = viscosity (Pas)
τ = Wall shear stress (Pa)
    At 37 °C
μ (Washed platelets in HEPES-Tyrode’s buffer with glucose) = 0.71 x 10^-3^ Pa·s
μ (Whole blood) = 3.8 x 10^-3^ Pa·s = 0.038 poise
μ (Water) = 0.695 x 10^-3^ Pa·s
1 centipoise = 1 mPa·s = 0.001 Pa·s 1 Pa = 10 dyn/cm^2^ = 1 Newton/m^2^
Shear Stress (Pa) = shear rate (cm/scm) × viscosity (Pa·s)
    $$Shear\;rate=\frac{shear\;stress}{viscosity}=\;\frac{Pa}{Pas}=s^{-1}$$
    **Table 1.** The flow rate of the platelet suspension required to achieve the desired shear rate and shear stress in the straight channel PDMS chip

Shear rate γ (s^-1^)	Shear stress τ (Pa)	Flow rate Q (μl/min)
150	0.1065	1.5
300	0.213	3
600	0.426	6
1,200	0.852	12
1,800	1.278	18
5,000	3.55	50
10,000	7.1	100
    **Figure 5.**
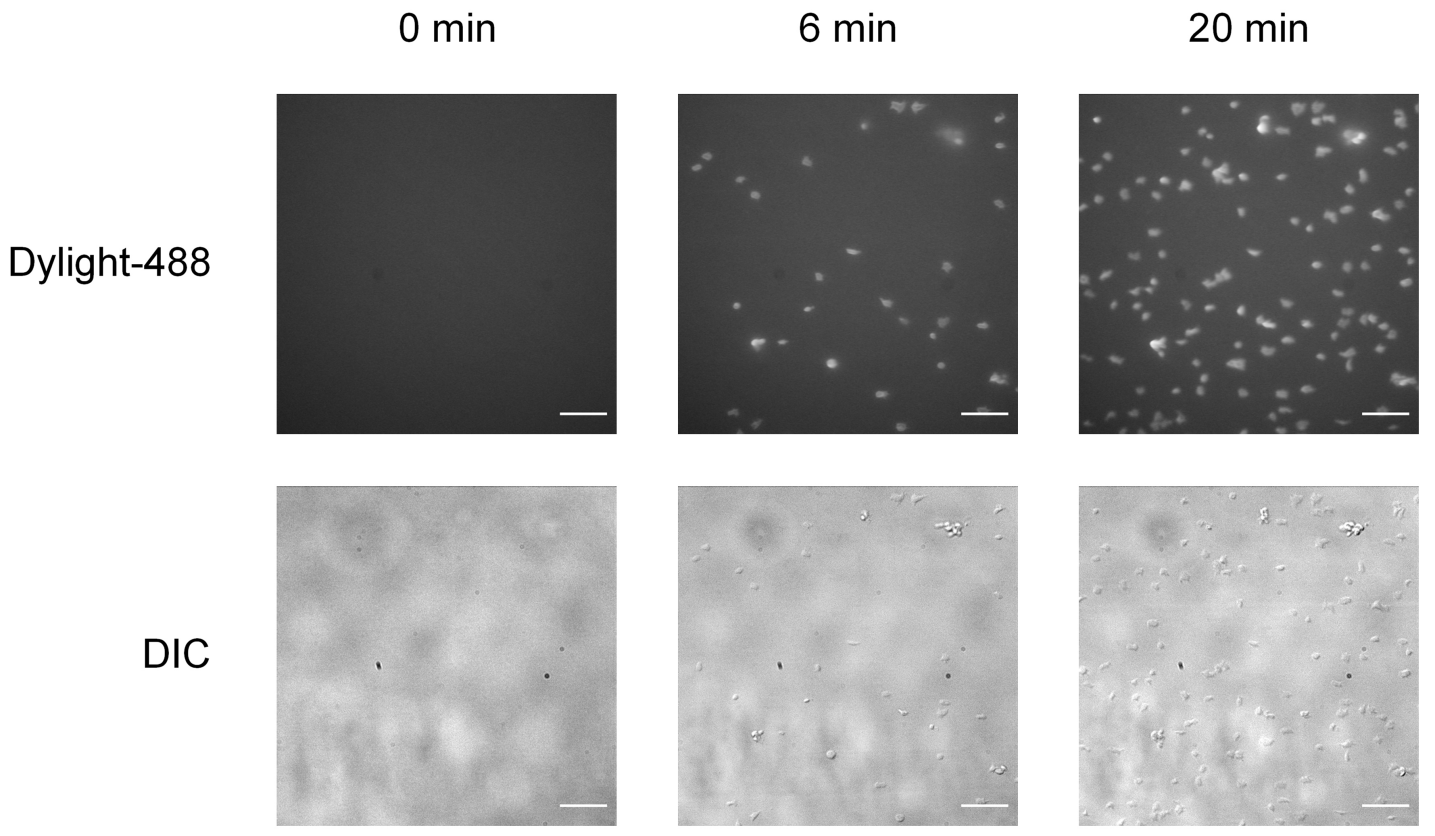
Adhesion of mouse platelets on fibrinogen under flow. Perfusion of platelets, labeled with anti-GPIb-488 antibody, on PDMS channels coated with fibrinogen (40 μg/ml) at a shear rate of 500 s^-1^. Time course of adherent platelets as captured by epi-fluorescence (top row) and DIC (bottom row) microscopy at 0, 6, and 20 min. Scale bars = 20 µm.
- Platelet adhesion using Vena8 Fluoro+ biochips
Vena8 Fluoro+ biochips are commercial biochips manufactured by Cellix^TM^. They are 8 channel biochips with a coverslip of 0.17 mm in width. The dimension of each channel is 0.1 mm in height, 0.4 mm in width and 25 mm length. Each channel on the biochip has a capacity of 8 microliters.
  1. Aspirate 10 μl fibrinogen (40 μg/ml in PBS pH 7.4) or vWF (100 μg/ml in PBS, pH 7.4) using a 20 μl pipette.
  2. Place the tip of the pipette into one opening of the channel of a Vena8 Fluoro+ biochip (Figure 6). Depress the plunger of the pipette to fill the channel with the coating substrate.
    **Figure 6.**
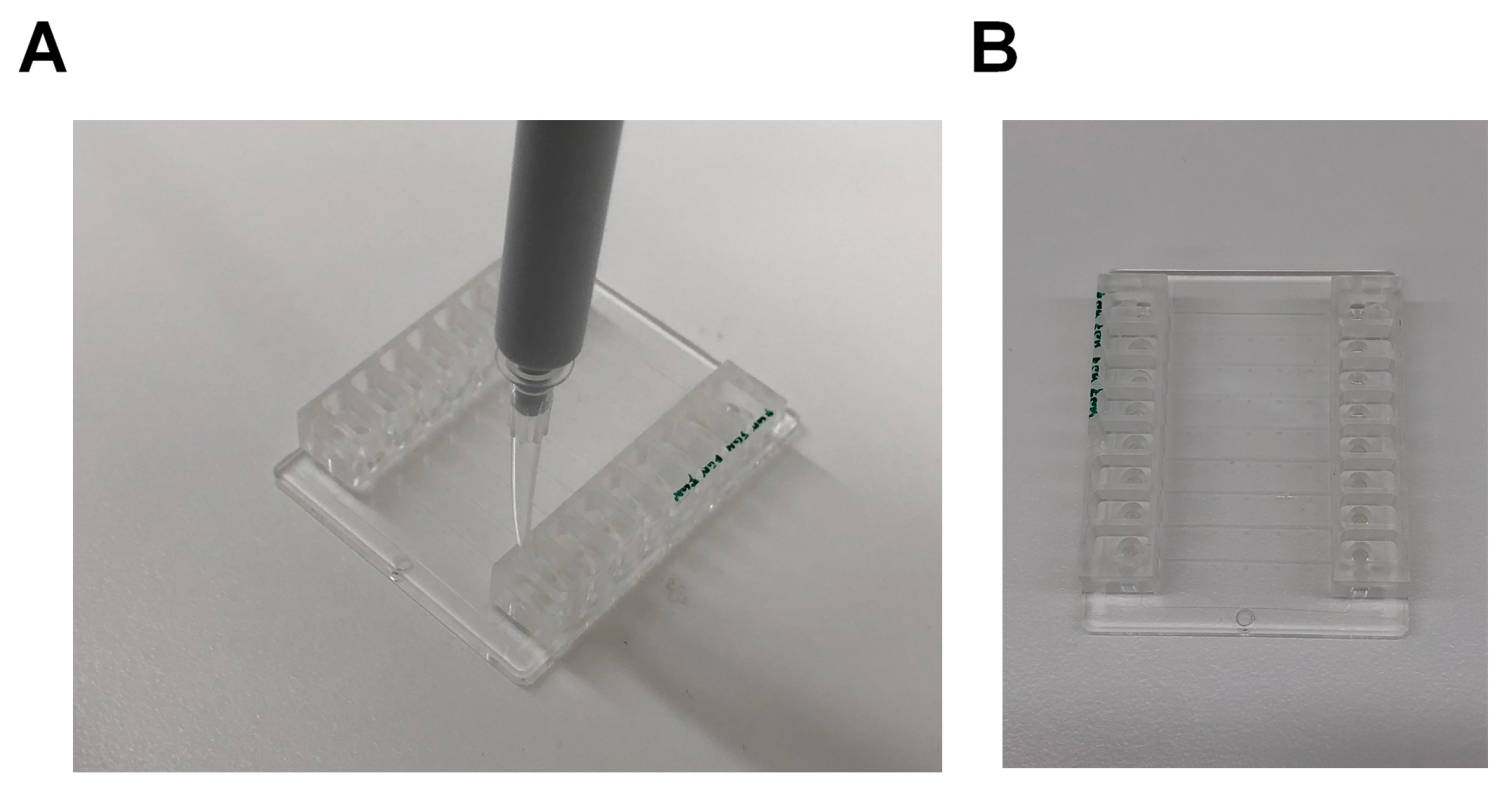
Coating of Vena8 Fluoro+ channels. A. A 10 μl pipette is placed into the opening of a channel and the channel was filled with the coating substrate. Once filled, the channel will be transparent. B. The first 4 channels of a biochip (marked green) are filled with coating substrate. The other 4 channels are empty, appearing grey.
  3. Place the biochip in a container with a tissue moistened with dH_2_O and close the container. Incubate the biochip overnight at 4 °C.
  4. Remove the coating substrate from the reservoir.
  5. Aspirate 10 μl of blocking buffer with a 10 μl pipette and fill the channel with the blocking buffer. Incubate the channel for 1 h at room temperature.
  6. Aspirate 10 μl of PBS with a 10 μl pipette and fill the channel to wash. Repeat Step F6 once.
  7. Connect the tubing to the biochip using the outlet pins.
  8. Submerge the tubing of one end of the channel in an Eppendorf tube filled with the washed platelets (Figure 7). Connect the tubing of the other end of the channel to the Mirus Evo Nanopump. Initiate the pump to draw the platelet suspension through the channel of the biochips. A table for the flow rates required to achieve the desired shear rate can be found at Cellix website under the technical specification. Alternatively, use the dimensions of the channel and the Formulas 1 and 2 in Procedure E to calculate the flow rate.
    **Figure 7.**
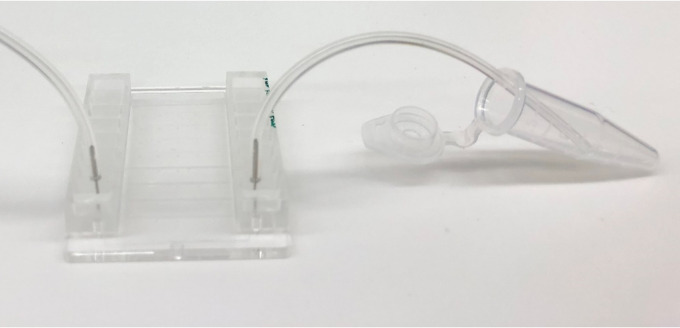
Setup of the Vena8 Fluoro+ biochip. An Eppendorf tube is used as a reservoir to hold the washed platelets. The other end of the channel is connected to tubing, which is attached to the Mirus Evo Nanopump.
- Image acquisition
  1. Assemble the syringe pump as shown below (Figure 8A): connect the inlet pin (1) (which is submerged in the Eppendorf containing platelet solution or whole blood) to the entry on one of the 8 channels on the chip. Connect the outlet pin (2) to the outlet of the channel and to the tubing (which is connected to the pump).
    **Figure 8.**
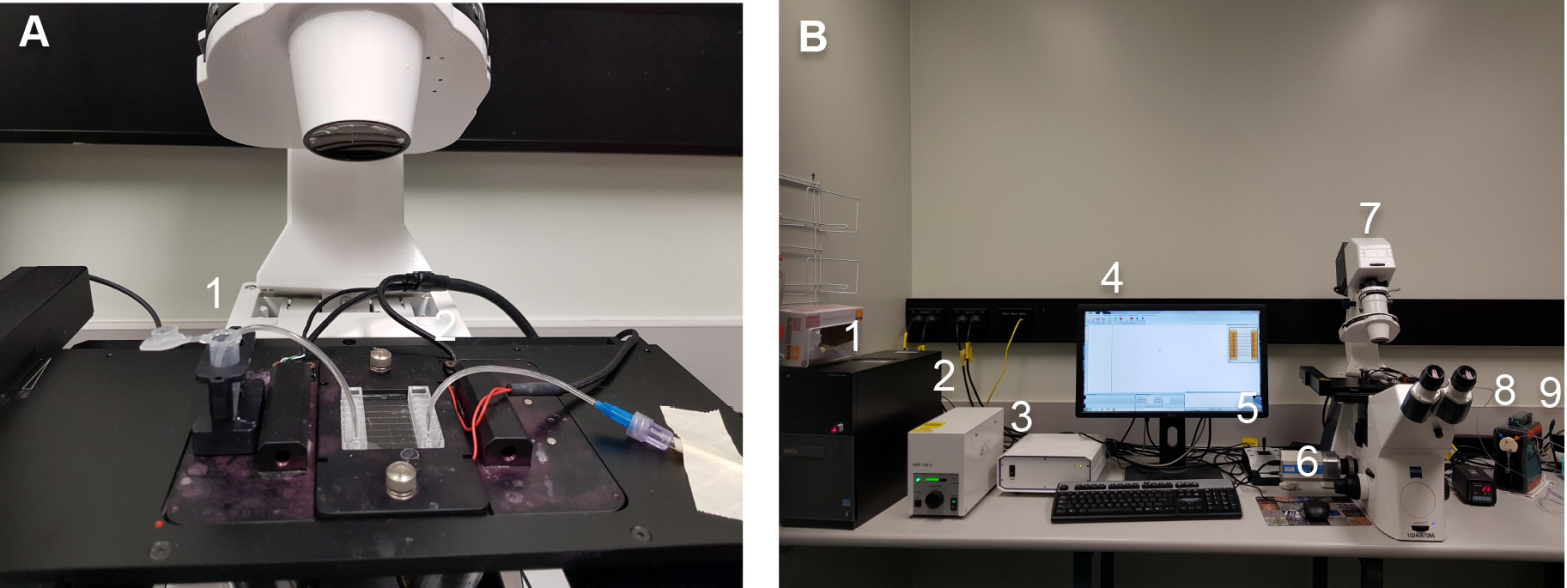
Assembly of the Cellix microfluidics system. A. Insertion of the Vena8 Fluoro+ biochip into the temperature-controlled stage holder. The inlet pin (1) is connected to the entry of one of the 8 channels on the chip. The outlet pin (2) is connected to the outlet of the channel and to the tubing leading to the pump. B. Setup of the Cellix microfluidics system. (1) Hard drive; (2) Lamp; (3) Motor control; (4) Screen display; (5) Stage control; (6) ExiBlu CCD camera; (7) Epifluorescence microsocope; (8) Temperature control; (9) Mirus Evo Nanopump.
  2. Switch the “Power on” button of the PC Hardrive (1), lamp (2), motor control (3), screen display (4), stage control (5), ExiBlu CCD camera (6), Epifluorescence microsocope (7), temperature control (8), Mirus Evo Nanopump (9) (Figure 8B). Initiate the VenaFlux Assayx64 software installed on the PC. This will bring up the Venoflux interface.
  3. Click on “new protocol’ or “open protocol” (if you have already set your parameters). Both options will bring up the same screen page (Figure 9A) with a list of commands. Each command is activated by right click of the mouse, which should be followed in the order displayed. The sequence of commands is: 1. VenFlux Setup, 2. Initialize VenFlux platform, 3. Start Video camera preview, 4. Geometry set up, 5. Update geometry, 6. Washout pump, 7. Washout cable, 8. Wash/connect biochip, 9. Washout chip, 10. Cell assay (Figure 9A). After the video camera preview has been initiated the channel will be displayed on the screen (Figure 9B).
*Notes:*
    - *For steps “washout pump” and “washout cable”, the outlet pin is removed. The pump flows the connected pump solution (water or PBS) into the cable whose free end should be placed into a waste collector.*
    - *For step “wash/connect biochip”, the outlet pin is connected to the cable but not to the outlet channel of the biochip. The needle of the outlet pin is held above the outlet position of the channel. When you right-click “wash/connect biochip”, the pump will deliver a squirt of 100 μl pump solution into the outlet well to avoid air entering into the channel when the needle is inserted into the outlet. This step can be omitted if you have already included some fluid in the well by pipetting.*
    - *For the step “washout chip”, the outlet pin has to be inserted into the outlet of the channel. The default setting is to deliver 40 μl of pump fluid at 1 μl/sec to wash out the channel.*
    - *For the step “cell assay”, enter the parameters for flow by adjusting the shear units, the acquisition time and capture delay (if required). After all steps have been entered, right click on “cell assay” to initiate the pump and acquire data. To stop the assay, click on “stop assay”.*
    - *To view the channel on the screen, switch the microscope connection to the camera. Switch to eye piece for fine focus of the channel.*
    - *
The steps involved in data acquisition (Figures 9-11) can be found in video webinars by Cellix, Ltd: Cellix Webinar:* *VenaFlux Platform Technical Presentation*;
*Cellix Webinar* : *Cellix Biochips with standard Syringe Pumps for Perfusion Assays*.
    **Figure 9.**
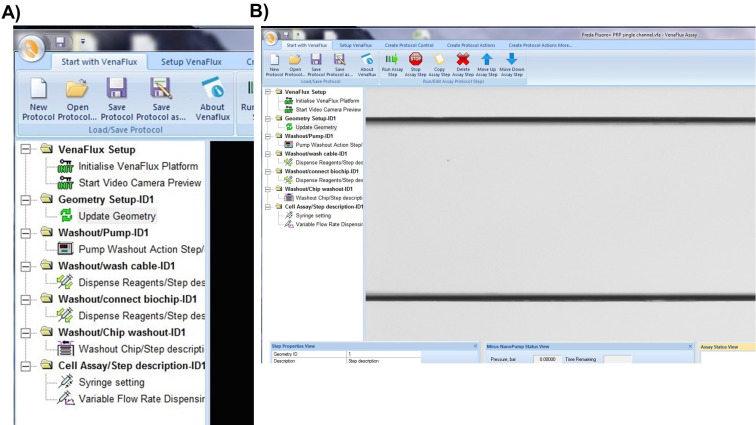
Initiating the VenaFlux Protocol. A. On the “Start with VenaFlux page” each command is activated by right click which should be followed in the order displayed. B. After the video camera preview has been initiated the channel will be displayed on the screen. The 2 horizontal black lines are the borders of the channel (20x magnification).
  4. The Vena8 Fluoro+ biochip has embedded markings to assist with the mapping of the biochip to the VenaFlux assay x 64 software. Each channel 1-8 has marked positions above channels 2, 3…6 which can be visualized under the microscope with 2, 3…6 dots respectively. On the dialogue screen of the VenaFlux assay x 64, at the top of the screen, click the option “Set up Venaflux”. This will bring another dialogue box displaying the x, y coordinates named “Set Vena8/VenaEC origin and channel/position spacing”. Click on Vena8 for biochip type. Using the joystick move the stage to focus on channel 1, position 2 (Figure 10A). Click “update position” on the left-top position. Using the joystick move the stage to channel 8, position 6 (Figure 10B). Click “update position” on the right-bottom position. Then press set. Coming back to the “Start with VenaFlux” page, you can click on the map of the biochip displayed at the right upper corner of the screen and this will automatically move the stage to the position.
*Note: There are no dots on the biochip for positions 1 and 8. The mapping of the Vena8 chip is based on positions 2 and 6, therefore the range of acquisition is smaller than the biochip’s dimensions.*
    **Figure 10.**
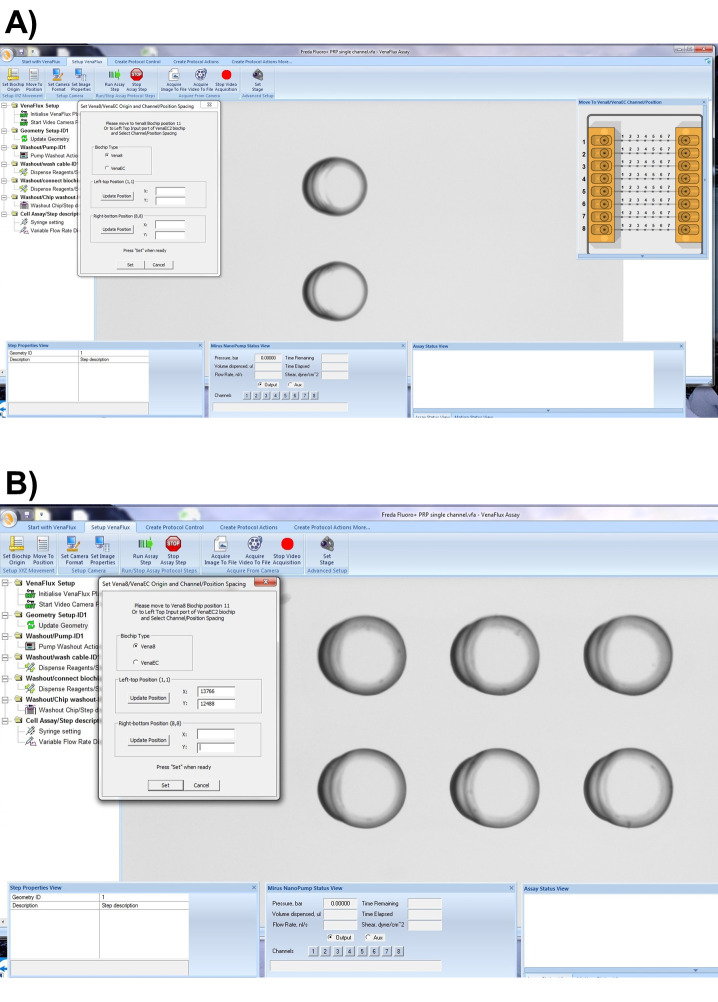
Mapping the Vena8 biochip. A. On the “Setup VenaFlux” page, move the stage to focus on channel 1, position 2 and click “update position” on the left-top position. B. Move the stage to channel 8, position 6. Click “update position” on the right-bottom position. Then press set.
  5. To start the assay, enter the desired parameters for flow by adjusting the shear units, the acquisition time and capture delay (Figure 11A). After all the steps have been performed, click on “start assay” to initiate the pump and data acquisition. To stop the assay, click on “stop assay”. To acquire an image, click on “acquire image to file” on the “Setup VenaFlux page”. This will save the image at the pre-specified location as a bitmap image (Figure 11B). To acquire a video, click on “acquire video to file”. The analysis of the images and image stacks of the videos can be performed by ImageJ as described below or using the ImagePro Premier 64-bit software. We prefer ImageJ for our analysis as it is flexible and can include adjustable macros.
    **Figure 11.**
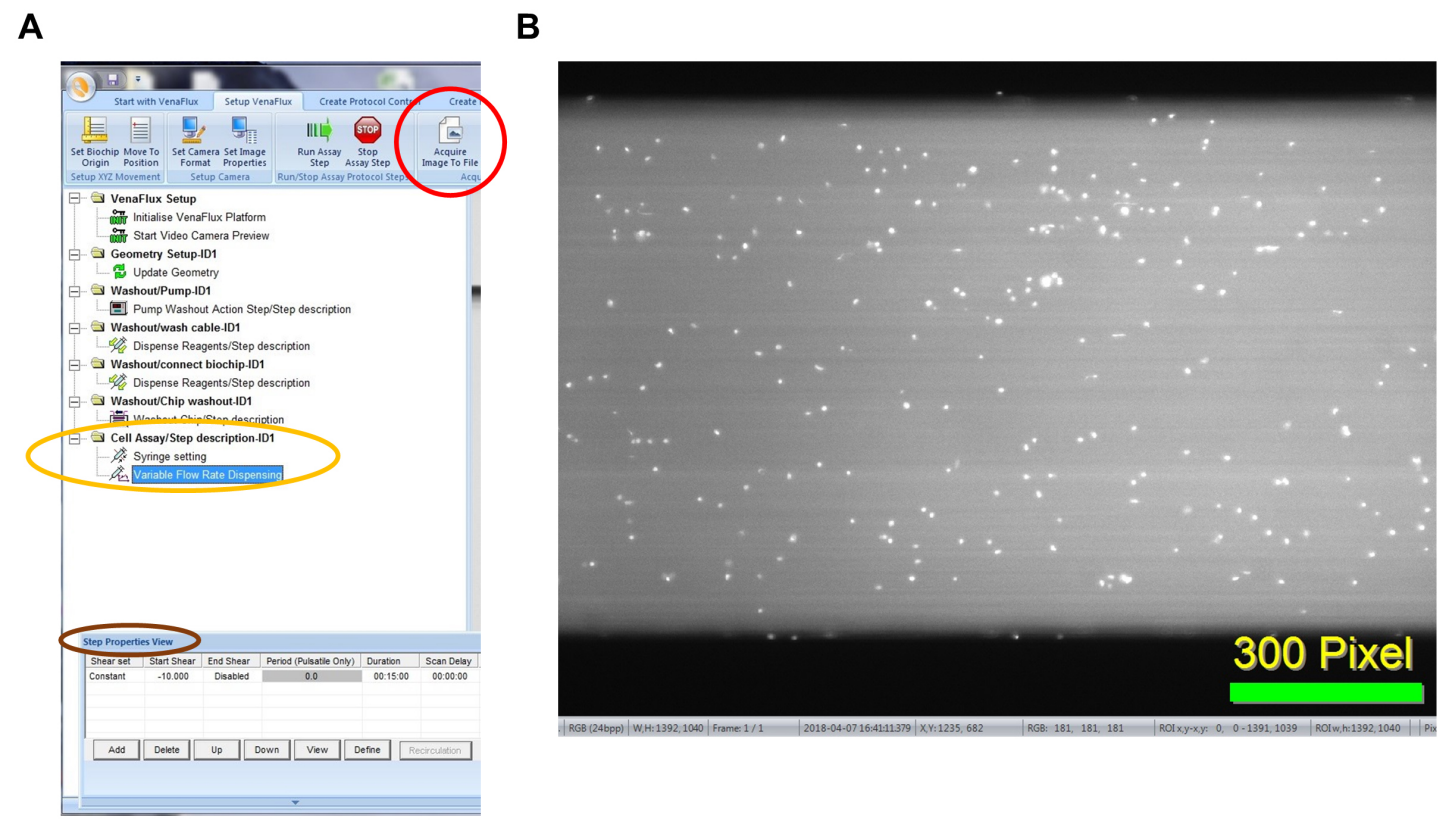
Initiating the VenaFlux Assay. A. Under “Cell assay” (circled in yellow), right click on the “variable flow rate dispensing” option and adjust the flow parameters on the “step properties view” icon (circled in brown). B. Click on “acquire image” (circled in red in panel A) to capture the image as bitmap file. Displayed is an example of an image captured of human platelets labeled with calcein 1 μg/ml (white dots) and perfused on a Vena8 channel coated with vWF 100 μg/ml.

## Data analysis

- PDMS biochips
  Two methods of manual analysis are described for the analysis of platelet adhesion over time: platelet counting and analysis by fluorescence intensity. This protocol describes the use of Fiji/ImageJ on PC for analysis, however the process is the same for Mac users.
***Platelet adhesion analysis by counting***
The steps involved in platelet count and platelet sum fluorescence data analysis (Figures 13-14) can be found in Video 3.
  **Video 3.**
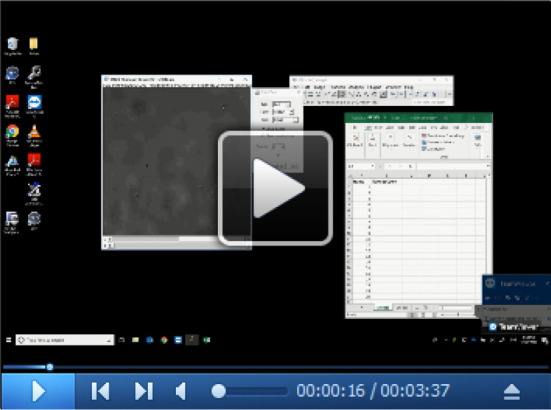
ImageJ analysis of platelet adhesion by counting
  1. Open Fiji/ImageJ version 1.52h.
  2. Click on the “Multi-point” tool on the tool bar. Double click on the “Multi-point” tool and on the “Point tool” window, check the “Label points” and “Show on all slices” checkbox (Figure 12).
    **Figure 12.**
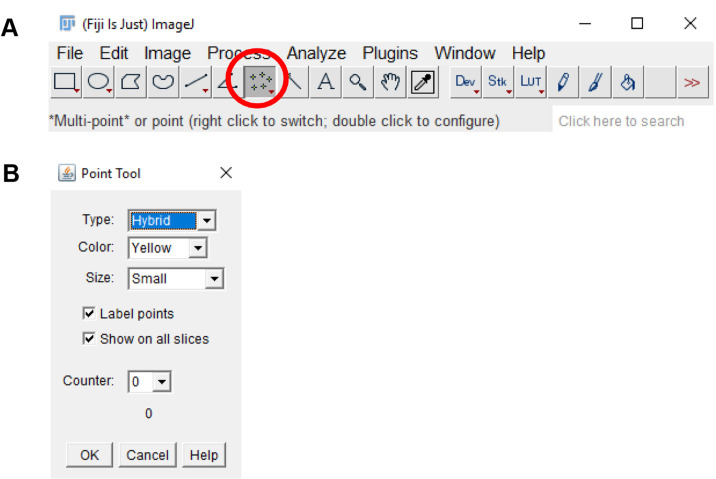
ImageJ multi-point tool. A. The multi-point tool is located on the toolbar of the ImageJ window (circled in red). B. Double-clicking the multi-point tool will open the point tool window. This window can be used to count placed markers and to adjust the display options of markers placed on the image.
  3. Click on every platelet in the field of view to count the number of adhered platelets (Figure 13). Verify the cell type by ensuring that the platelets are ve+ stained on the 488 nm channel.
    **Figure 13.**
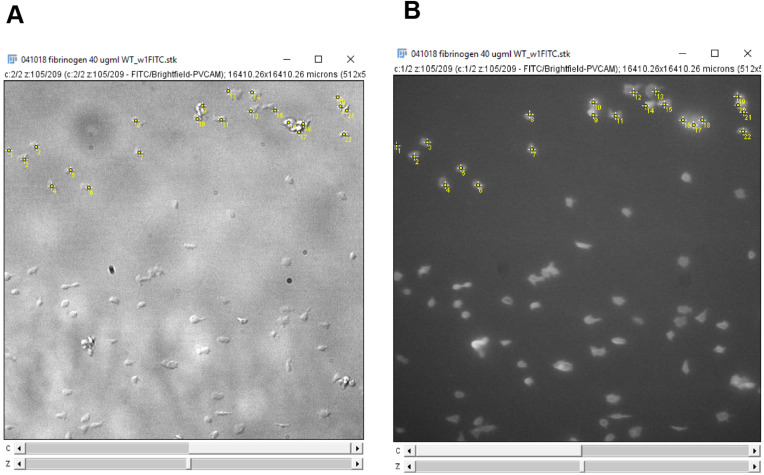
Counting platelets using the ImageJ multi-point tool. A. Platelets are counted on the DIC channel. B. The platelets are verified using the fluorescence channel to check for anti-GPIb-488 staining. Clicking on an image, with the multi-point tool selected, will leave a marker with a number on the image. Holding “alt” and then clicking on a marker will remove the marker. Each subsequent click or removal will increase or decrease the number on the marker by one respectively. Use this feature to count the number of platelets on the image. Alternatively, after clicking on each platelet on the image, use the counter on the point tool window (Figure 12) to determine the number of platelets counted.
  4. Enter the platelet count onto GraphPad prism or Microsoft Excel.
  5. Move to the next frame on the image stack, click on additional platelets that have adhered and de-select platelets no longer present as described in step 3. Record the platelet counts (Figure 13) and repeat for each frame in the image stack.
  6. Plot the platelet count against time (Figure 14).
    **Figure 14.**
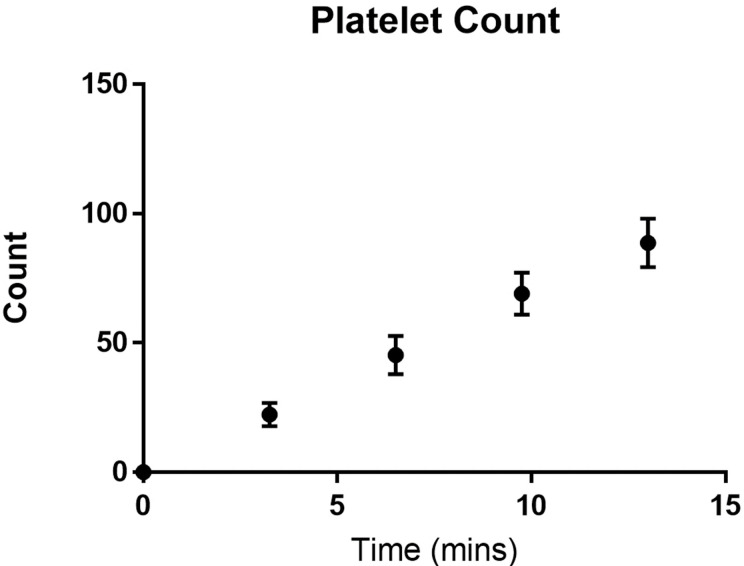
Number of platelets adhered to a fibrinogen channel over time. Wild type C57BL/6 mouse platelets were washed and flowed across PDMS biochips coated with fibrinogen 40 μg/ml at a shear rate of 500 s^-1^. The platelet count in the field of view was determined every 3 min. The total number of adherent platelets was plotted against time using GraphPad prism. Dots represent the mean platelet count at the specific time point and the error bars represent the standard error of mean from n = 3 experiments.
***Platelet adhesion analysis by fluorescence intensity analysis***
The steps involved in platelet sum fluorescence data analysis (Figures 15-22) can be found in Video 4.
  **Video 4.**
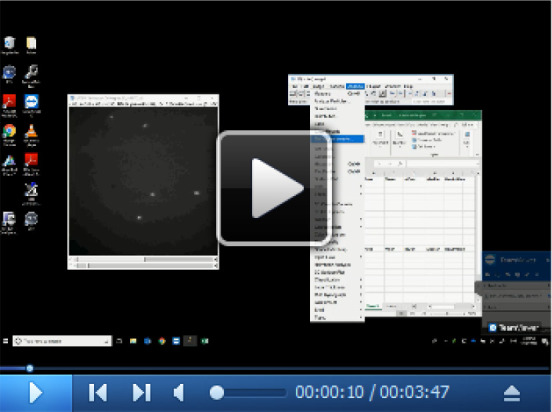
ImageJ analysis of platelet adhesion by fluorescence intensity
  1. Open Fiji/ImageJ.
  2. Click the “Analyze” tab and select “Set Measurements” (Figure 15).
    **Figure 15.**
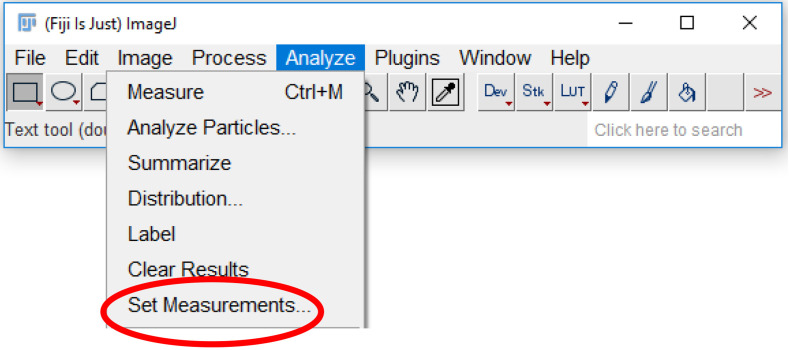
Accessing analysis parameters. The parameters that will be displayed on a measurement readout can be selected from the set measurements menu. To access the menu, select the “analyze” tab (highlighted in blue), and click “Set measurements…” (circled in red).
  3. Check the “Area”, “Mean gray value”, “Integrated density”, and “Median” checkboxes (Figure 16).
*Note: Each of these measurements provides a description of the platelets. For single platelets, “Area” describes the area taken up by the region of interest drawn around platelets. “Mean gray value” and “Median” are both methods of quantifying the mean fluorescence intensity of a single platelet or platelet aggregate. “Integrated density” quantifies the total intensity for a selected area.*
    **Figure 16.**
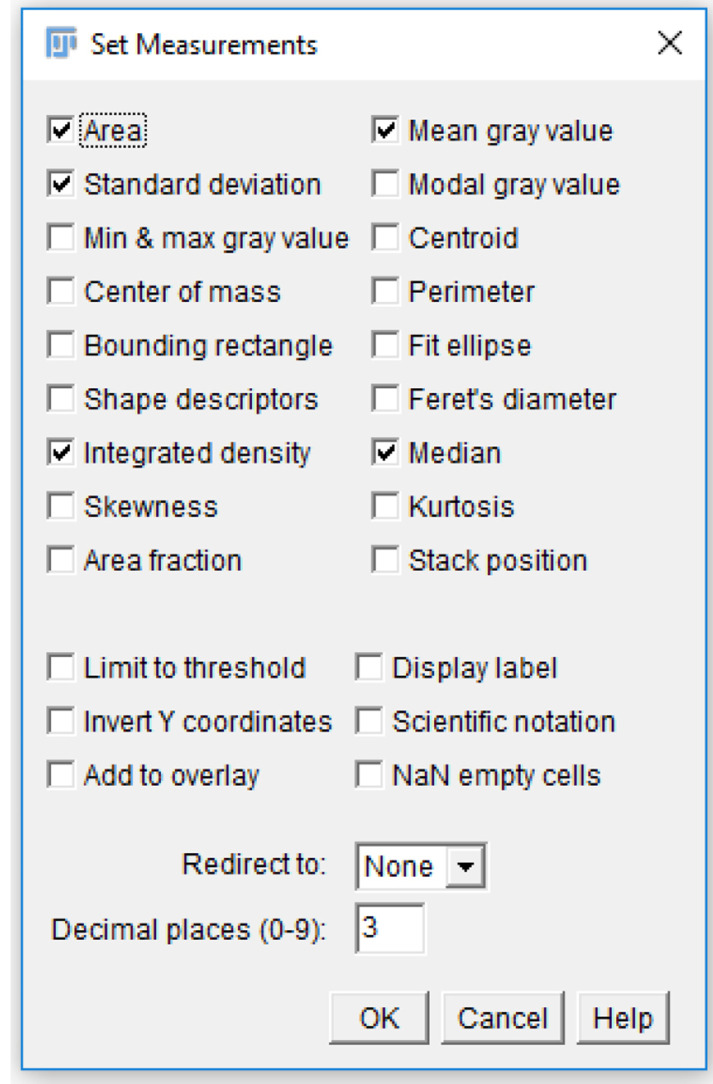
Analysis parameters used for measuring platelet fluorescence intensity. Various parameters can be selected for measuring fluorescence in a region of interest. For the analysis of platelet fluorescence intensity in this protocol, we have selected “Area”, “Standard deviation”, “Mean gray value”, and “Integrated density”.
  4. Click the “Analyze” tab, click “Tools”, and select “ROI manager…” (Figure 17).
    **Figure 17.**
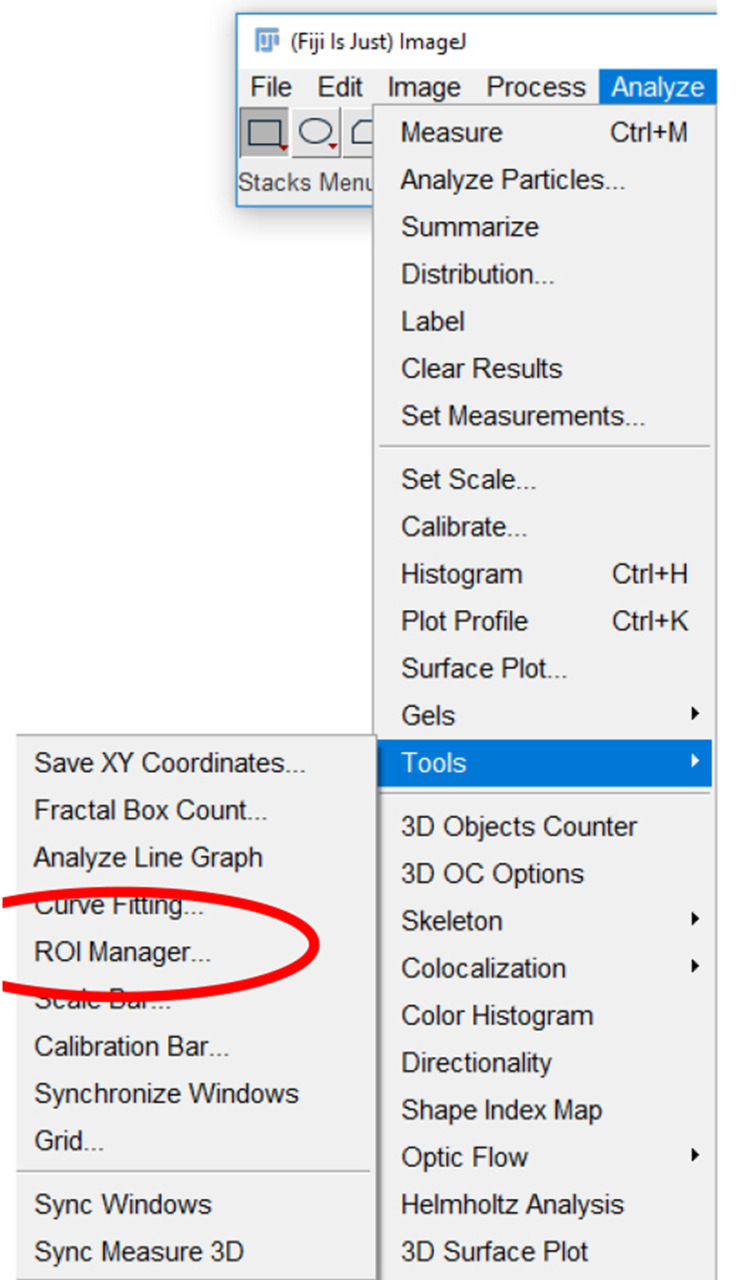
Region of Interest (ROI) manager on ImageJ. The ROI manager can be accessed by selecting “Analyze” and then “Tools” (highlighted in blue) and clicking on “ROI manager…” (circled in red).
  5. Click on the “Freehand selections” tool on the tool bar (Figure 18).
    **Figure 18.**
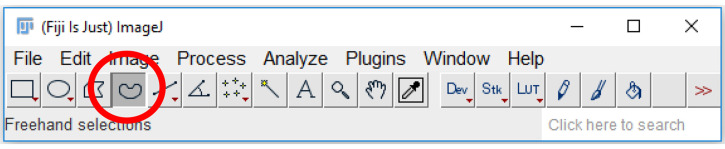
Freehand selections tool on ImageJ. The freehand selection tool (circled in red) can be used to trace a region of interest around a platelet or platelet aggregate. The region of interest can then be stored on the ROI manager, and the parameters, set in Step B3, can be measured.
  6. For each frame on the time stack, on the 488 nm channel, circle a platelet or platelet aggregate and click “Add” on the ROI manager window (Figure 19).
    **Figure 19.**
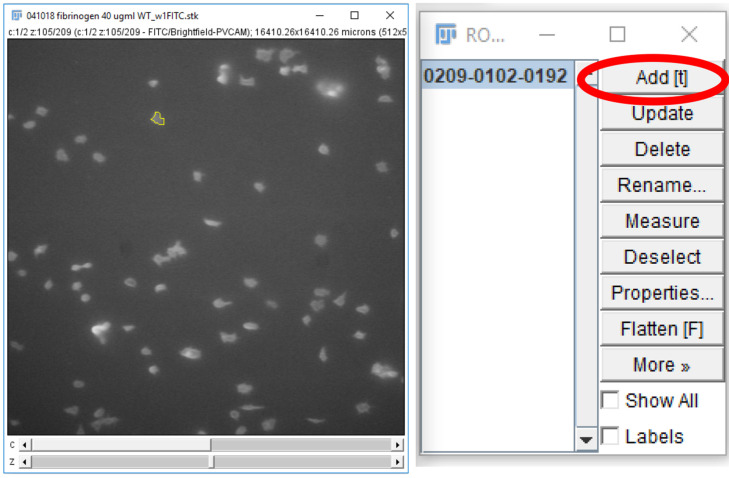
Storing regions of interest on the ROI manager. A region of interest, drawn using the freehand selections tool, can be stored on the ROI manager either by pressing “t” on the keyboard or clicking “Add” on the ROI manager (circled in red).
  7. Click and drag the region of interest to an area without platelets near the original position (Figure 20) and click “Add” on the ROI manager window. This measures the background fluorescence.
    **Figure 20.**
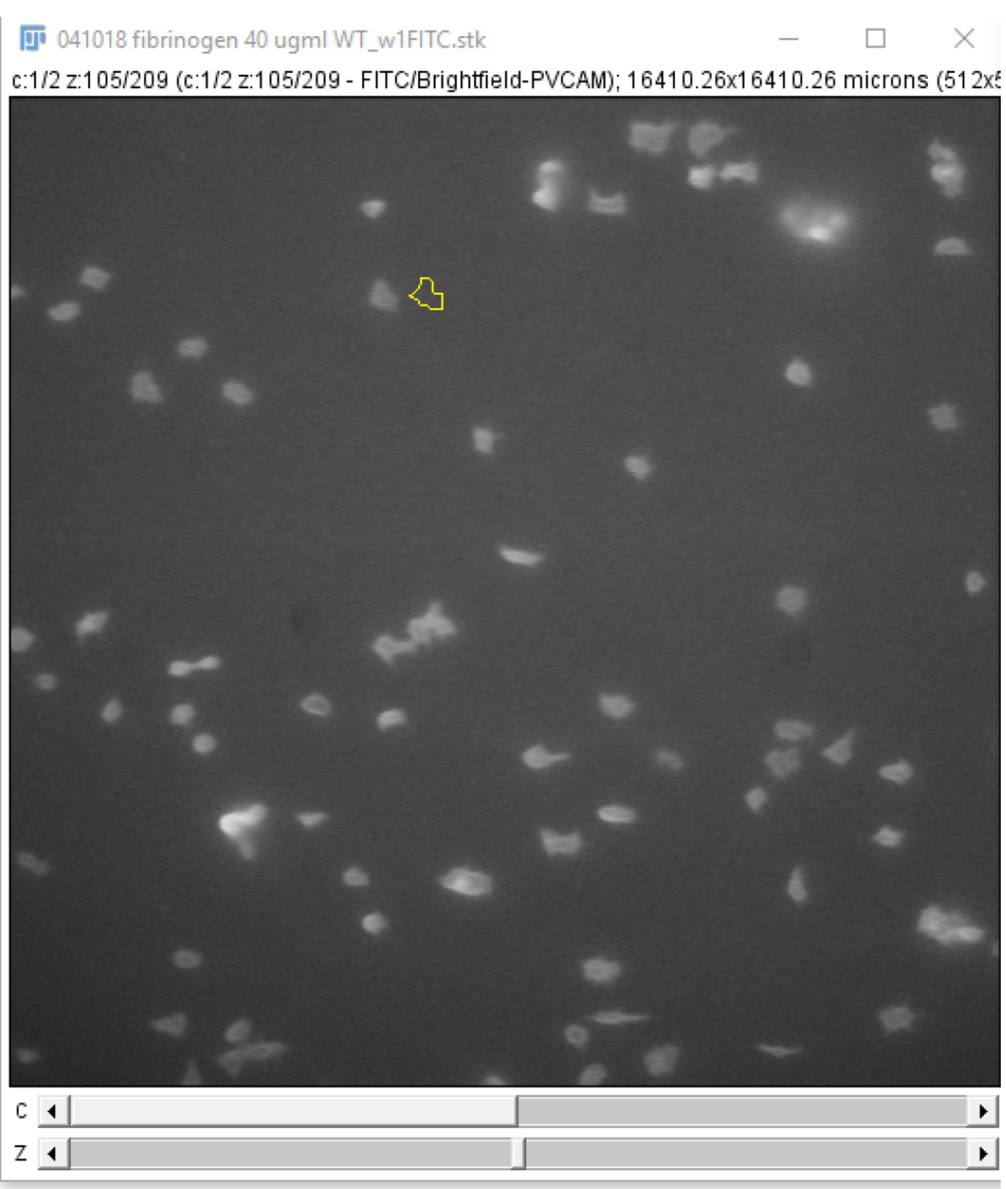
“Background” region of interest. The region of interest drawn in Step B6 can be used for selecting a new region of interest to subtract background fluorescence intensity measurements. Click and drag the region of interest away from the platelet/platelet aggregate and add the new area to the ROI manager.
  8. Select both regions on the ROI manager window and click “Measure” (Figure 21).
    **Figure 21.**
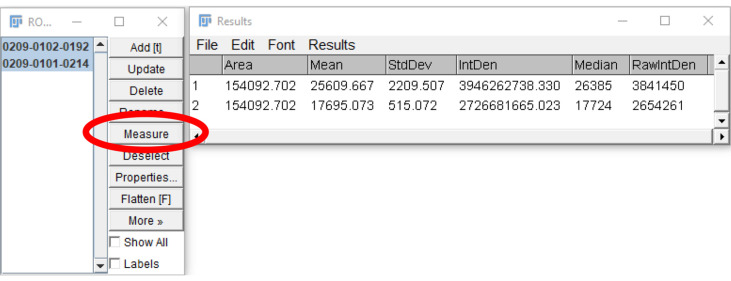
Measurement of regions of interests. After the desired regions of interest are selected (highlighted in blue), and “Measure” is clicked (circled in red), a new window will open, showing the measurements of the parameters selected in Step B3.
  9. Subtract the Mean/Median/RawIntDen of the background fluorescence measurement from the corresponding platelet measurement. Enter the data onto GraphPad prism or Microsoft Excel.
  10. Repeat steps 5-9 for each platelet and platelet aggregate in the frame, sum the fluorescence, and record the value. Repeat for all frames in the image stack.
  11. Plot the fluorescence intensity over time on GraphPad prism or Microsoft Excel (Figure 22).
    **Figure 22.**
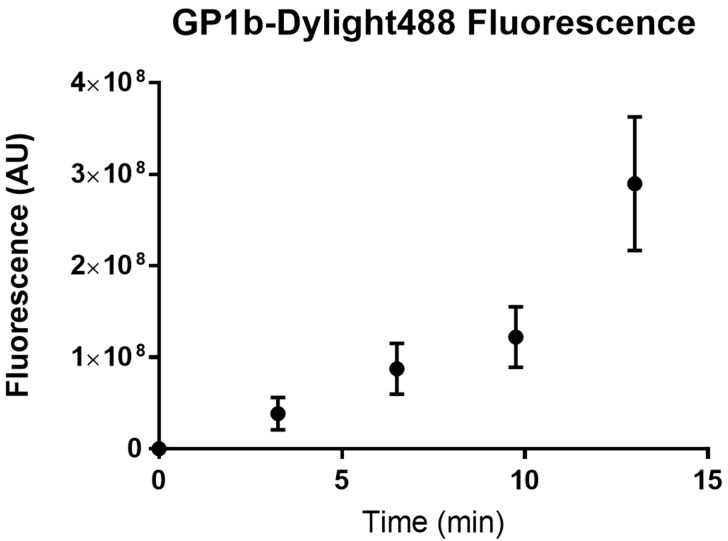
Fluorescence of platelets adhered to a fibrinogen channel over time. Wild type C57BL/6 mouse platelets were washed and flowed across PDMS biochips coated with fibrinogen 40 μg/ml at a shear rate of 500 s^-1^. The sum of platelet fluorescence in the field of view was determined every 3 min. The sum of platelet fluorescence was plotted against time using GraphPad prism. Dots represent the mean sum of fluorescence at the specific time point and the error bars represent the standard error of mean from n = 3 experiments.
***Statistical analysis of the change in platelet adhesion over time***
  1. Open GraphPad Prism.
  2. Select the “grouped” tab.
  3. Check the “enter _____ replicate values in side-by-side columns”, entering the maximum number of replicates performed in the experiment.
  4. In the column titles, enter the time points that are to be analyzed. Make sure that the time between each point is equal.
  5. Enter the experimental data under the corresponding time points on the first row.
  6. Click “Analyze” and under the “Column analyses” drop down menu, select “One-way ANOVA (and non-parametric)”.
  7. Choose analysis parameters based on the desired analysis:
    - For data that have a Gaussian distribution, under the “Assume Gaussian distribution?” section, click “Yes, use ANOVA” option (Notes 1 and 2).
    - For data that do not have a Gaussian distribution, *e.g.*, data that are skewed in distribution, under the “Assume Gaussian distribution?” section, click “No, use nonparametric test” (Notes 1 and 2).
  8. Select “Ok”, and under the “Results” drop-down menu to the left of the page, select the analysis for the data and check the *P*-value. A *P*-value < 0.05 indicates statistical significance.
***Statistical analysis of treatment effect on platelet adhesion at a specific time point***
  1. Open GraphPad Prism.
  2. Select the “grouped” tab.
  3. Check the “enter _____ replicate values in side-by-side columns”, entering the maximum number of replicates performed in the experiment.
  4. In the column titles, enter the titles of the treatments that are to be analyzed.
  5. Enter the data of the treatments at a specific time point in the first row.
  6. Click “Analyze” and under the “Column analyses” drop-down menu, select “*t-*test (and nonparametric tests)”.
  7. Choose the analysis parameters based on the desired analysis:
    - For data that have a Gaussian distribution, under the “Assume Gaussian distribution?” section, click “Yes, use parametric test” option (Notes 1 and 2).
    - For data that do not have a Gaussian distribution, *e.g.*, data that are skewed in distribution, under the “Assume Gaussian distribution?” section, click “No, use nonparametric test” (Notes 1 and 2).
- Commercial biochips
Images are captured using the accompanying VenaFlux 2.3 imaging software and videos are captured at a rate of 1 frame per 5 s (30 frames). Images are analyzed at positions 2, 4 and 6 (located at 6, 14 and 22 mm from the entry site of the platelet suspension) of the channels. These positions are representative of flow nearest, mid-way and furthest from the entry of the platelet suspension into the channel. The platelet count and sum of platelet fluorescence are measured at 3-minute intervals using Image J or the ImagePro Premier 64-bit software http://www.mediacy.com/imagepro. Data are exported into Excel or GraphPad Prism for statistical analysis.

## Recipes

1. 1x PBS
  137 mM NaCl
  2.7 mM KCl
10 mM Na_2_HPO4
1.8 mM KH_2_PO4
  pH 7.4
*Note: Prepare in advance and store at room temperature for up to 1 year.*
2. 10% Extran
40 ml Extran^®^ MA 02
360 ml ddH_2_O
  pH 7.0
*Note: Prepare fresh extran 10% dilution before use and discard after use.*
3. Blocking buffer
  2% bovine serum albumin
  1x phosphate buffered saline
  pH 7.4
*Note: Prepare albumin 2% solution in advance and store in aliquots at -20 °C until use for up to 1 year. Avoid freeze-thawing aliquots more than 2 times.*
4. HEPES-Tyrode’s buffer with glucose
  20 mM HEPES
  134 mM NaCl
0.34 mM Na_2_HPO_4_
  2.9 mM KCl
12 mM NaHCO_3_
1 mM MgCl_2_
1 mM CaCl_2_
  5 mM D-glucose, added prior to washing platelets
  pH 7.4
*Note: Prepare the HEPES-Tyrode’s buffer in advance (without the D-glucose) and store at room temperature for up to 1 year. Add the D-Glucose to an aliquot of the HEPES-Tyrode’s buffer and use within 24 h.*
